# A randomized controlled trial of sequential vs simultaneous use of Foley balloon catheter and oxytocin for induction of labor in nulliparous pregnant women

**DOI:** 10.1016/j.xagr.2023.100252

**Published:** 2023-08-01

**Authors:** Delayehu Bekele, Mariamawit Asfaw, Balkachew Nigatu, Birhanu Kebede, Lemi Belay Tolu, Abdulfetah Abdulkadir Abdosh, Abraham Fessehaye Sium

**Affiliations:** Department of Obstetrics and Gynecology, St. Paul's Hospital Millennium Medical College, Addis Ababa, Ethiopia

**Keywords:** FOIL-N, Foley balloon catheter, induction in Ethiopia, induction of labor, simultaneous vs sequential Foley and oxytocin

## Abstract

**BACKGROUND:**

Although recent evidence suggests the simultaneous approach use of oxytocin for induction of labor in nullipara, there is limited data from low-income settings that support this.

**OBJECTIVE:**

This study aimed to determine whether induction of labor with simultaneous use of oxytocin and a Foley balloon catheter decreases the induction of labor to delivery interval in nulliparous women, compared with sequential use of a Foley balloon catheter followed by oxytocin.

**STUDY DESIGN:**

This was a randomized controlled trial of nulliparous women with singleton pregnancies presenting for induction of labor at >28 weeks of gestation at St. Paul's Hospital Millennium Medical College (Addis Ababa, Ethiopia). The participants were randomly assigned to either the simultaneous group (the use of oxytocin and a Foley balloon catheter for induction of labor) or the sequential group (overnight intracervical Foley balloon catheter placement followed by the use of oxytocin the next morning). The primary outcome was induction of labor to delivery interval. Comparisons between the groups were made using the Student *t* test or Wilcoxon rank-sum test and chi-square test on Stata (version 15; StataCorp LLC, College Station, TX). This study is registered with the Pan African Clinical Trials Registry (identifier: PACTR201709002509200).

**RESULTS:**

From November 2019 to March 2020, a total of 140 women were randomly assigned to the simultaneous group (70 women) or the sequential group (70 women). The median oxytocin initiation to delivery intervals were 6.09 hours (range, 4.03–10.7) in the sequential group and 8.1 hours (range, 4.7–11.6) in the simultaneous group (*P*=.46). The mean Foley balloon catheter insertion to delivery intervals were 16.09±5.7 hours in the sequential group and 8.06±4.2 hours in the simultaneous group (*P*<.001). Cesarean delivery rate, composite neonatal outcomes, and chorioamnionitis were not different between the 2 groups.

**CONCLUSION:**

In nulliparous pregnant women, induction of labor using the simultaneous approach did not shorten the oxytocin initiation to delivery interval compared with the sequential approach. Moreover, both approaches showed no difference in the rates of adverse maternal and neonatal outcomes.

**AJOG MFM at a Glance**

**Why was this study conducted?**

The current suggestion of using oxytocin and a Foley balloon catheter for induction of labor (IOL; simultaneous) in nulliparas is not supported by data from low-income settings. This study aimed to investigate whether the simultaneous use of oxytocin and a Foley balloon catheter for IOL in nullipara reduces the IOL to delivery interval compared with the sequential approach.

**Key findings**

Compared with the sequential approach, the simultaneous use of oxytocin and Foley balloon catheter insertion did not shorten the IOL to delivery interval in nulliparous women. Moreover, the sequential and simultaneous groups showed no difference in composite maternal and neonatal adverse outcomes.

**What does this add to what is known?**

Our study supports the previously suggested practice of IOL for nulliparas. Although simultaneous use of oxytocin with Foley balloon catheter insertion does not shorten IOL to delivery interval in nulliparous women, the simultaneous approach can be safely practiced in a low-income setting with comparable outcomes with the sequential approach.

## Introduction

Since the 1980s, several studies have proven that a transcervical Foley balloon catheter is an effective induction of labor (IOL) agent that improves initial cervical examination and achieves vaginal delivery rates comparable with other induction techniques, including vaginal prostaglandins.^1^ The Foley balloon catheter ripens the cervix directly through mechanical dilation of the cervical canal and indirectly by increasing endogenous prostaglandin secretion.^2^^,^^3^ The advantages of Foley balloon catheter insertion compared with pharmacologic methods include being of low cost compared with some drugs, low risk of tachysystole, few systemic side effects, and convenient storage requirements.^4^ The most important concern with leaving the catheter is infection and sepsis, and this is if it is left for more than 24 hours. However, research findings do not support this concern, even in the presence of intrauterine fetal demise.^5^, ^6^ A randomized trial that compared 4 IOL methods—misoprostol alone, Foley balloon catheter alone, misoprostol-cervical Foley balloon catheter concurrently, and Foley balloon catheter-oxytocin concurrently—found no difference in the occurrence of chorioamnionitis in the 4 methods.^7^

Until recently, definite answers have not been found on whether a Foley balloon catheter with simultaneous oxytocin could improve the efficacy of induction of labor (IOL) outcome, compared with the sequential use of a Foley balloon catheter followed by oxytocin initiation for IOL 12 hours later or Foley balloon catheter expulsion, whichever comes first. Moreover, there is no strong evidence from low-income settings on this topic. The results of a recent meta-analysis support the practice of Foley balloon catheter insertion with simultaneous use of oxytocin for IOL in nulliparas. The study included 5 randomized controlled trials (RCTs) conducted in high-income settings, which compared the simultaneous group with the sequential group in terms of IOL to delivery interval, and 1 RCT conducted in a low-income setting but done with a different objective of comparing the effectiveness of a Foley balloon catheter plus oxytocin initiation for IOL with that of oxytocin initiation for IOL alone.^8^

In addition to the possibility of shortening the IOL to delivery interval, the simultaneous use of oxytocin with Foley balloon catheter insertion for IOL may reduce hospital stay length by eliminating the need for overnight stay of patients in a hospital for cervical preparation. It may decrease the use of hospital resources, including labor and delivery staff's time, intake of intravenous fluids, and patient overall cost. These factors have a paramount significance in low-income settings, such as in St. Paul's Hospital Millennium Medical College (Addis Ababa, Ethiopia). Intracervical Foley balloon catheter placement for cervical preparation during IOL is standard practice at our hospital. We use the sequential method (overnight intracervical Foley balloon catheter placement followed by oxytocin initiation for IOL 12 hours later the next morning). Done with the objective of determining whether IOL with the simultaneous use of oxytocin and Foley balloon catheter decreases the IOL to delivery interval in nulliparous women, compared with the sequential use of a Foley balloon catheter followed by oxytocin, our study presents robust evidence on simultaneous vs sequential Foley balloon catheter insertion to oxytocin initiation during IOL in a low-income setting.

## Materials and Methods

### Study design

This was a 2-arm RCT of nulliparous women with singleton pregnancies presenting for IOL >28 weeks of gestation at St. Paul's Hospital Millennium Medical College in Addis Ababa, Ethiopia, from November 2019 to March 2020. Before initiation of the trial, the protocol was approved by the relevant ethics committees and regulatory agencies of Ethiopia, the institutional review board of St. Paul's Hospital Millennium Medical College, and the Ethiopian Science and Technology Commission. No formal interim analysis was planned or conducted.

### Participants

We approached nulliparous pregnant women with singleton pregnancies presenting for IOL for different indications and those who volunteered to participate in the study, and after ethical clearance was secured, we enrolled these women against the inclusion and exclusion criteria of the study. The inclusion criteria were nulliparous women at 28 weeks of gestation; women with a live, nonanomalous singleton fetus in vertex presentation; women with an initial cervical dilation of ≤2 cm; women who were admitted for IOL for indications determined by their primary provider; and women in whom cervical ripening with Foley balloon catheter was planned. The exclusion criteria were patients with previous uterine surgery, patients with unexplained vaginal bleeding, patients with any history of latex allergy, and patients with any contraindication to vaginal delivery. Data were double entered into EpiData Manager (version 4.6.0.0; USA, Cali). Study supervisors regularly checked the completeness of CRFs and data encoding. The data were exported into Stata (version 15; StataCorp LLC, College Station, TX) for cleaning and analysis.

### Randomization and masking

After signing the informed consent, the participants were randomized using previously prepared envelopes. The randomization scheme was prepared before the start of the study by an individual who was not part of the study team. A permuted block of differing sizes of 6 to 10 was used and was prepared using a computer-based random number generator. Cards allocating patients to either the simultaneous group or synchronous group were placed in sealed, opaque, numbered envelopes. Moreover, the participants were given the next envelope from the consecutively numbered envelopes. After enrollment, each patient had a transcervical Foley balloon catheter placed in a standard, sterile fashion. A 16F 30-mL Foley balloon catheter was used in each case and inflated to 60 mL with normal saline. Proper placement of the Foley balloon catheter was confirmed by digital examination in all patients. The end of the Foley balloon catheter was taped to the patient's inner thigh.

For those patients assigned to the simultaneous group, oxytocin initiation for IOL was started within 1 hour of insertion of the Foley balloon catheter and titrated according to our department's protocol. Oxytocin infusion was started at 2.5 mU/min. This dose was doubled every 30 minutes according to the department's protocol. Fetal heart rate (FHR) and contraction patterns were monitored every 15 minutes in patients receiving oxytocin. For patients assigned to the sequential group, oxytocin initiation for IOL was started within 1 hour of spontaneous expulsion of the Foley balloon catheter using the aforementioned institutional protocol. If spontaneous expulsion did not occur, the Foley balloon catheter was removed after 12 hours of cervical ripening. For all patients, the remainder of the patient's labor was managed by the primary provider following standard obstetrical practice.

### Procedures

An orientation was given to the labor and delivery staff and residents regarding the trial and the criteria for eligibility. Eligible women were identified by our staff in the waiting area for priming and IOL cases, before any intervention for IOL was started, and the identified women were informed about the research and enquired about their interest in hearing more about this study. Interested patients were approached by study personnel and counseled on the risks and benefits of participation. They were informed that their participation would be voluntary and that choosing not to participate in the study would not affect their care. Data were collected through abstraction and interviews by trained research midwives using a structured questionnaire, which was completed throughout the hospital stay. The following information were included in the questionnaire: maternal personal and demographic data (age and address), gestational age, indication for IOL, Bishop score, mode of delivery, duration of IOL, chorioamnionitis, and newborn information (Apgar score, weight, sex, and neonatal intensive care unit [NICU] admission).

### Outcomes

The primary outcome of this study was the IOL to delivery interval, defined as the time elapsed between the initiation of the first dose of oxytocin and the delivery of the baby (“baby out time”). The secondary outcomes were hospital stay (Foley balloon catheter insertion to delivery), cesarean delivery (CD) rate, uterine tachysystole, chorioamnionitis, failed IOL, and a composite neonatal outcome. The composite neonatal score was defined as having at least 1 of the following neonatal adverse outcome indicators: 5-minute Apgar of <5, NICU admission, or neonatal death, specifically derived from iterative assessments and used in care comparisons and outcome predictions.^9^ Failed IOL was defined as failure to initiate good uterine contraction and was diagnosed if adequate uterine contractions were not achieved after 6 to 8 hours of oxytocin administration and use of the maximum dose for at least 1 hour.

### Statistical analysis

**Figure 1:**
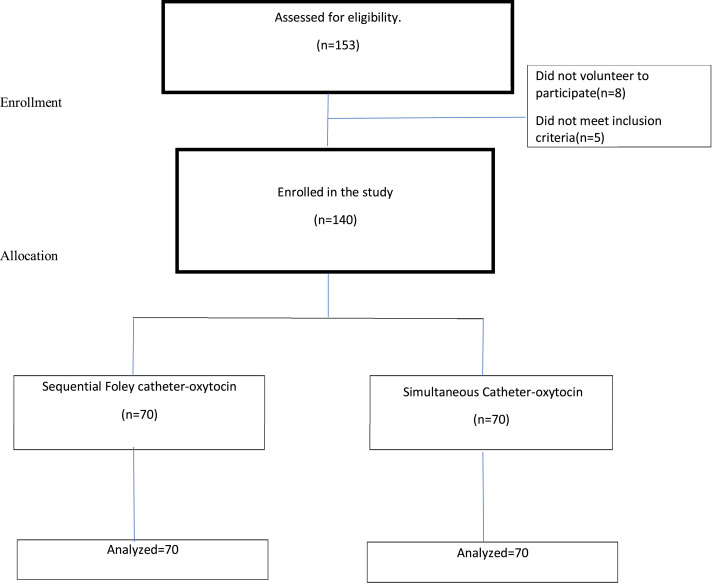
CONSORT flow chart

Comparing 2 means using OpenEpi (version 3.03; USA, Cali) and considering a 95% confidence interval (CI), power of 80%, a 1:1 ratio of sample size of group 2 to group 1, a mean difference to detect at least 0.5 hour and standard deviations (SDs) from the only previous similar study were used.^9^ A sample size of 35 patients in each group was needed to detect at least a 0.5-hour difference in time to delivery. To see the difference in only those who have vaginal deliveries, the sample size was recalculated taking into consideration the IOL success rate in our hospital, which was 54% as documented in a previous study conducted in our hospital.^10^ Hence, a sample of 66 was calculated to get 35 vaginal deliveries. An additional 5% of patients were planned to be recruited to account for study dropout, incomplete medical records, or inadvertent study crossover. A total of 140 cases, 70 from each group, were recruited (Figure 1). An intention-to-treat analysis was performed. The mean and SD or median and interquartile range (IQR) are presented for quantitative variables, and frequency and percentage are presented for qualitative variables, with comparisons between randomization groups using the Student *t* test or Wilcoxon rank-sum test and chi-square test, respectively. All hypothesis tests were 2-sided and performed at the 5% level of significance. Analyses were performed using STATA (version 15; StataCorp LLC, College Station, TX).

Bekele. Labor induction in nulliparas: sequential vs simultaneous use of Foley balloon and oxytocin. Am J Obstet Gynecol Glob Rep 2023.

### Role of the funding source

Apart from allocating a financial grant to support the conduct of this study, the funder (Center for International Reproductive Health Training) had no input into the study design, data interpretation, review, and approval of this report. The corresponding author had full access to all the data in the study and had final responsibility for the decision to submit for publication.

## Results

There was no difference in maternal age and body mass index between the sequential and simultaneous groups (Table 1). The Bishop score and indications for IOL were not different between the 2 groups, except for the gestational age. The mean gestational age was lower by 0.85 weeks in the simultaneous group.

**Table 1:** Baseline characteristics of the sequential and simultaneous groups in Ethiopia, 2019–2020

Characteristic	Sequential group (n=70)	Simultaneous group (n=70)	*P* value
Maternal age, mean±SD	24.93	34.99	.94
Gestational age, mean±SD	39.46±2.16	38.61±2.52	.0358
Initial Bishop score, median (IQR)	3.0 (2.0–5.0)	4.0 (3.0–6.0)	.081
Body mass index, mean±SD	24.5 (3.3)	23.5 (3.0)	.080
Indication for IOL, n (%)			
After term	10 (14)	11 (16)	1.00
Gestational hypertension	5 (7)	4 (6)	1.00
Preeclampsia	27 (39)	31 (44)	.61
IUGR	24 (34)	16 (23)	.19
Oligohydramnios	38 (54)	39 (56)	1.00

*IOL*, induction of labor; *IQR*, interquartile range; *IUGR*, intrauterine growth restriction; *SD*, standard deviation.

The medians and IQRs of oxytocin initiation to delivery interval were 6.9 (IQR, 4.3–10.7) in the sequential group and 8.1 (IQR, 4.7–11.6) in the simultaneous group (Table 2). There was no difference in the oxytocin initiation to delivery interval between the 2 groups (*P*=.46). This remained nonsignificant when the analysis was performed for only those delivered vaginally. The Foley balloon catheter insertion to delivery interval was almost half in individuals in the simultaneous group compared with individuals in the sequential group and was statistically different between the 2 groups (8.6 hours in the simultaneous group vs 16.9 hours in the sequential group; *P*<.001).

**Table 2:** Foley balloon catheter insertion to delivery interval and oxytocin initiation to delivery interval of the sequential and simultaneous groups in Ethiopia, 2019–2020

Variable	Sequential group (n=70)	Simultaneous group (n=70)	*P* value
Oxytocin initiation to delivery interval, median (IQR)	6.9 (4.3–10.7)	8.1 (4.7–11.6)	.46
Oxytocin initiation to vaginal delivery interval, median (IQR)	6.9 (4.6–8.7)	7.0 (4.3–10.9)	.78
Foley balloon catheter insertion to delivery interval, mean (SD)	16.9 (5.7)	8.6 (4.2)	<.001

*IQR*, interquartile range; *SD*, standard deviation.

When it comes to maternal and neonatal outcomes, the rate of CD was 57% (40/70) in the sequential group, which was not statistically different from 69% (48/70) in the simultaneous group (*P*=.16) (Table 3). Most patients in the 2 groups were high-risk patients (in which case providers lowered their threshold for CD intervention), and the fact that we do not determine scalp pH after the detection of nonreassuring FHR (NRFHR) patterns (eg, category 2) may explain this high rate of CD among the participants.

**Table 3:** Maternal and neonatal outcomes of the sequential and simultaneous groups in Ethiopia, 2019–2020

Variable	Sequential group (n=70)	Simultaneous group (n=70)	*P* value
CD	40 (57.0)	48 (69.0)	.16
Indication for CD			
Failed IOL	12 (30.0)	11 (22.9)	.44
NRFHR	22 (55.0)	24 (50.0)	
Meconium-stained liquor	4 (10.0)	9 (18.8)	
Others (such as CPD)	2 (5%)	4 (8.3%)	
Uterine atony	0 (0)	1 (1.4)	1.00
Chorioamnionitis	1 (1.4)	0 (0)	1.00
Uterine tachysystole	5 (7.1)	0 (0)	.027
Birthweight (g)	2813.57±525.91	2655.71±556.55	.087
Composite neonatal score	12 (17.1)	16 (22.9)	.40
Neonatal death	2 (2.9)	1 (1.4)	.54
NICU referral	10 (14.3)	15 (21.4)	.38
Low Apgar score (<5)	2 (2.9)	1 (1.4)	1.00

Uterine tachysystole is defined as >5 contractions per 10 minutes, averaged over 30 minutes.

*CD*, cesarean delivery; *CPD*, cephalopelvic disproportion; *IOL*, induction of labor; *NICU*, neonatal intensive care unit; *NRFHR*, nonreassuring fetal heart rate.

NRFHR was the most common indication for CD, and the rate of CD was not different between the 2 groups. Similarly, there was no statistically significant difference in the rates of uterine atony, chorioamnionitis, and composite neonatal outcomes between the 2 groups. There were 5 cases of uterine tachysystole in the sequential group, but there was no case of uterine tachysystole in the simultaneous group, which was statistically significant (*P*=.027). All tachysystole cases occurred after oxytocin initiation for IOL, not with the Foley balloon catheter insertion alone. There was no case of postpartum hemorrhage in the 2 groups.

## Comment

### Principal findings

In this RCT, nulliparous women with singleton pregnancies who were allocated to the simultaneous use of oxytocin with Foley balloon catheter insertion group (simultaneous group) and those who were allocated to the sequential use of oxytocin for IOL after an overnight cervical ripening with Foley balloon catheter insertion group (sequential group) did not have different IOL to delivery intervals. There was no difference in maternal and neonatal outcomes between the 2 groups.

### Results

Several studies have aimed to study whether Foley balloon catheter and oxytocin should be used simultaneously or sequentially for IOL. A recent meta-analysis of 6 RCTs reported that there is no statistically significant difference in delivery within 24 hours between Foley balloon catheter insertion with simultaneous use of oxytocin and sequential Foley balloon catheter insertion followed by oxytocin initiation for IOL 12 hours later (relative risk [RR], 1.09; 95% CI, 0.92–1.29). There was no difference in maternal and neonatal outcomes between the 2 groups, including CD, chorioamnionitis, and low Apgar score. Nevertheless, the analysis of women according to parity showed that Foley balloon catheter insertion with simultaneous use of oxytocin for IOL in nulliparous women was associated with a significant increase in delivery within 24 hours (RR, 1.32; 95% CI, 1.12–1.55).^8^ An RCT of Foley balloon insertion for IOL in multiparas reported that simultaneous use of oxytocin and Foley balloon catheter insertion for IOL does not decrease time to delivery in multiparas.^9^ Here, the median oxytocin initiation to delivery intervals were 6.9 hours in the sequential group and 8.1 hours in the simultaneous groups, which are not statistically different (*P*=.46). There was no statistically significant difference in the rates of CD, uterine atony, chorioamnionitis, and composite neonatal outcomes between the 2 groups. However, the rate of uterine tachysystole was higher in the sequential group (5 cases in the sequential group vs no case in the simultaneous group). This is contrary to reports of previous studies, which showed no difference between the 2 groups.^11^^,^^12^ The prolonged stay of a Foley balloon catheter in situ might result in a release of endogenous prostaglandins that increase the odds of tetanic contraction. Nevertheless, all uterine tachysystole cases occurred after the start of oxytocin for IOL, which makes it difficult to ascribe this complication to the difference in the timing of Foley balloon catheter insertion.

Despite not being a primary objective of the study, a shorter Foley balloon catheter insertion to delivery interval in the simultaneous group than in the sequential group (8.6 hours in the simultaneous group vs 16.9 hours in the sequential group; *P*<.001) is an important finding in our study. Studies on outpatient Foley balloon catheter insertion for IOL presented conflicting results of hospital stay duration. A 2021 RCT found that the duration of hospital stay was significantly shorter in the outpatient group: 35.8±20.2 hours in the outpatient group vs 45.2±16.2 hours (*P*=.001).^13^ This is contrary to the findings of another RCT published in 2018, which reported that outpatient cervical ripening in parous women does not shorten the time from labor ward admission to delivery if oxytocin is initiated simultaneously with inpatient transcervical catheter placement.^14^ Another RCT demonstrated that, in nulliparous patients undergoing elective IOL at term, outpatient cervical ripening with a transcervical Foley balloon catheter reduced the time from admission to delivery. However, admissions before scheduled IOL were higher in the outpatient group (22% vs 5%; RR, 4.7; 95% CI, 1.4–15.4).^15^

### Clinical implications

Our study shows that simultaneous use of oxytocin with Foley balloon catheter insertion can be safely practiced for IOL in nulliparous women in a low-income setting with comparable outcomes with that of the sequential approach, although it does not shorten IOL to delivery interval in nulliparous women. As available evidence on outpatient Foley balloon catheter insertion remains controversial, simultaneous use of oxytocin and Foley balloon catheter insertion could be an alternative solution to tackle the problem of bed shortage for IOL rampant in low-income settings, as it avoids hospital bed occupancy for overnight cervical priming with Foley balloon catheter insertion. In our hospital, the common practice for IOL is the sequential method (overnight intracervical Foley balloon catheter placement followed by oxytocin initiation for IOL the next morning).

### Research implications

Our study shows that simultaneous use of oxytocin with Foley balloon catheter insertion and sequential Foley balloon catheter insertion followed by oxytocin initiation for IOL in nulliparous pregnant women have comparable IOL to delivery intervals. Future RCTs should compare simultaneous Foley balloon catheter insertion vs oxytocin initiation for IOL vs outpatient overnight Foley balloon catheter insertion followed by oxytocin initiation for IOL the next morning in low-income settings in terms of rates of achieving vaginal delivery within 24 hours.

### Strengths and limitations

The strengths of our study include appropriate sample size allocation, low-income study setting, and analysis of Foley balloon catheter insertion to delivery interval. The main limitation of our study is the lack of analysis of the delivery rate within 24 hours, which is explained by the protocol of IOL in our setting, which sets a 12-hour cutoff time to declare failed IOL. Another limitation of this study is not including the analysis of total hospital stay. Future studies should compare simultaneous use of oxytocin and Foley balloon catheter vs outpatient Foley balloon catheter for IOL in nulliparous women.

### Conclusions

Our findings demonstrate that simultaneous initiation of oxytocin with Foley balloon catheter insertion and the sequential approach have comparable oxytocin initiation to delivery intervals. This may have implications for solving bed shortage for IOL in low-income settings that is associated with an overnight admission of patients for cervical preparation with a Foley balloon catheter.
